# Could the Hemodynamic Progression of Aortic Valve Stenosis be Slowed Pharmacologically? The Unsolved Dilemma

**DOI:** 10.31083/RCM26537

**Published:** 2025-02-12

**Authors:** Gloria Santangelo, Francesco Antonini-Canterin, Pompilio Faggiano

**Affiliations:** ^1^Department of Cardio-Thoracic-Vascular Diseases, Foundation IRCCS Ca’Granda Ospedale Maggiore Policlinico, 20122 Milano, Italy; ^2^Department of Cardiology, ORAS Hospital, 31045 Motta di Livenza (Treviso), Italy; ^3^Fondazione Poliambulanza, Cardiovascular Department, 25124 Brescia, Italy

## 1. Introduction

Aortic stenosis (AS) is one of the most prevalent valvular heart diseases, affecting nearly 10% of the elderly population [1]. It is a progressive disease, and its rapid progression has been associated with a poor prognosis [2]. Given the complexity of the underlying pathological process, extensive research has focused on identifying pharmacological interventions that could prevent or slow aortic valve degeneration. However, despite these efforts, no drug has been shown to effectively halt the disease’s progression or prevent aortic valve calcification (AVC) to date [3].

## 2. AS, Diabetes Mellitus and Dyslipidemia

Histopathological evidence suggests that degenerative AS is an active process, sharing numerous similarities with atherosclerosis, including lipid accumulation, inflammation, and calcification. Diabetes mellitus (DM) is an independent risk factor for the development and progression of AVC. Patients with both AVC and DM may benefit from undergoing aortic valve replacement (AVR) earlier compared to those without DM [4]. The accelerated atherosclerosis observed in diabetic patients is largely driven by a combination of factors, including endothelial dysfunction, abnormal platelet activity, altered smooth muscle behavior, disrupted lipoprotein metabolism (particularly low-density lipoprotein—LDL), and coagulation abnormalities [5]. The link between LDL cholesterol (LDL-C) levels and degenerative AS is well established. Research shows that lipids, particularly oxidized LDL, play a key role in triggering cell signaling pathways that leads to heart valve calcification. Oxidized LDL has been detected in calcified valves, highlighting its role in the process [6]. This connection is further supported by the presence of widespread atherosclerotic lesions in the aortic valves of patients with familial hypercholesterolemia, even in the absence of other atherosclerotic risk factors [7].

## 3. DM and AS: Epidemiological Evidence

Although the connection between DM and degenerative aortic AS has been observed, studies have reported varying prevalence rates of DM in patients with degenerative AS. Some studies indicates that DM is a significant comorbidity in a substantial portion of individuals with degenerative AS, with rates as high as 41% in DM patients, while other studies estimate a lower prevalence of around 30% [8, 9]. Conversely, one notable finding from the SALTIRE (Scottish Aortic Stenosis and Lipid Lowering Trial, Impact on Regression) trial was that only about 5% of patients with AS had DM. In a 2003 study by Ortlepp *et al*. [10], 20% of patients with severe AS were diabetic, compared to 18% in age-matched controls. This variability in reported prevalence may stem from differences in study populations, methodologies, diagnostic criteria, or underlying patient characteristics, making it difficult to draw definitive conclusions about the exact relationship between DM and AS. Alternatively, larger cohort studies have indicated that the prevalence of DM is significantly elevated in individuals with AS. For instance, the Multi-Ethnic Study of Atherosclerosis (MESA), involving 5723 participants, demonstrated that DM increased the likelihood of developing AS (odds ratio (OR) 2.06; 95% CI, 1.39–3.06) [11]. Additionally, the Cardiovascular Health in Ambulatory Care Research Team (CANHEART) conducted a population-based observational study on a cohort of 9.8 million adults. After a median follow-up of 13 years, the study concluded that DM was linked to an increased risk of AS (OR 1.49; 95% CI, 1.44–1.54) [12]. Therefore, there seems to be a lack of consensus on the proportion of DM among both the general population and those with degenerative AS.

## 4. Results of the Present Study

The study by He *et al* [13]. aimed to evaluate the progression of AS using echocardiography in 170 patients with mild to severe AS between January 2015 and December 2020 at a single center. Patients were stratified into tertiles based on LDL-C levels and type 2 DM status. The study found that the risk of rapid AS progression was highest in patients with type 2 DM and LDL-C levels of 3.14 mmol/L or higher. The findings highlight that poorly managed LDL-C in patients with type 2 DM can accelerate the progression of AS, underscoring the importance of aggressive LDL-C management in these groups.

## 5. AS and Imaging

In this study, the AS progression rate was primarily evaluated using echocardiography ultrasound. Echocardiography is the standard method for detecting and assessing the severity of AS. However, in addition to ordinary evaluation of left ventricular (LV) hypertrophy and ejection fraction, early assessment of LV deformation parameters, particularly global longitudinal strain, along with myocardial fibrosis, as estimated by cardiac magnetic resonance imaging, could significantly enhance the decision-making process. Indeed, the presence of myocardial fibrosis is associated with worse survival following AVR. This is particularly important in diabetic patients, where symptoms may be subtle or less apparent [14]. Patients with severe AS who are asymptomatic are also at increased risk of cardiac events or sudden cardiac death [15]. Furthermore, in specific subgroups such as patients with low-flow low-gradient or paradoxical low-gradient AS, determining disease severity can be challenging, which may lead to delayed treatment and poorer outcomes. In these cases, aortic valve calcium scoring via computed tomography (CT) serves as a valuable complementary tool, especially when echocardiographic findings are unclear or conflicting. The aortic valve calcium burden quantified by CT is a strong predictor of AS progression and clinical events, including the need for AVR and death [16].

AS and coronary atherosclerosis share many clinical risk factors, with coronary artery disease (CAD) being a negative prognostic factor in AS patients. Consequently, coronary arteries evaluation through invasive coronary angiography or coronary CT angiography is recommended when planning for surgical or transcatheter AVR [17]. Recently, increased attention has been given to moderate AS [18], especially when accompanied by LV dysfunction. In diabetic patients, who often have multivessel calcific CAD and sometimes LV dysfunction, it is reasonable to anticipate a rapid progression of moderate AS, suggesting the need for an early therapeutic intervention.

## 6. AS Medical Management

The major practical implication of the He *et al*. [13] study is the suggestion that aggressive management of LDL-C in patients with type 2 DM may be crucial in slowing AS progression. This contrasts with the 2020 clinical guidelines for the management of patients with valvular heart disease, which do not recommend statins to slow or limit AS progression due to insufficient evidence [19]. The guideline position is supported by data from a meta-analysis involving 2344 AS patients treated with statin versus placebo, which found no significant improvement on slowing AS progression, AVR rates, or cardiovascular mortality [20]. On the other hand, Antonini-Canterin *et al*. [21] emphasize the distinction between AS, primarily characterized by calcium accumulation, and aortic sclerosis, characterized by lipid deposits. These two conditions have distinct pathological features and progress at different rates. In fact, AS is linked to calcium deposition, while aortic sclerosis is a condition that involves lipid deposition and thickening of the valve without significant obstruction of blood flow. Although less severe than AS, aortic sclerosis can progress to AS over time. The study suggests that statins may play a beneficial role in slowing the progression of early aortic valve lesions, particularly in cases of aortic sclerosis where lipid deposition plays a more significant role. The effectiveness of statins in this context likely stems from their ability to lower LDL-C and reduce lipid accumulation, thereby mitigating early disease progression. Lee *et al*. [22] showed that aortic valve calcium, as measured by CT, is linked to coronary atherosclerosis driven by calcified plaque, while non-calcified plaques, which are more related to coronary events, showed no such link. While statins effectively reduce coronary plaque, particularly non-calcified vulnerable plaques, they do not slow the progression of aortic valve calcium, suggesting distinct biological pathways for AS and CAD. In statin-naïve patients, statins reduce coronary atherosclerosis by targeting lipid-rich plaques, but in statin-treated patients, this benefit does not extend to AVC. Thus, despite shared risk factors like elevated LDL-C, the distinct mechanisms of calcification in AS and plaque formation in CAD explain why statins are more effective for coronary disease than for AS progression.

## 7. Lipoproteinp(a) [Lp(a)]

He *et al*. [13] did not report any data about lipoprotein(a) [Lp(a)]. Measurement of Lp(a) should be considered at least once in a person’s lifetime, to identify individuals with elevated (>50 mg/dL) or extremely high levels (>180 mg/dL). These individuals face a lifetime risk of atherosclerotic cardiovascular disease comparable to that of heterozygous familial hypercholesterolemia [23]. Elevated Lp(a) levels significantly increase the risk of future cardiovascular diseases [24, 25], and recent studies have highlighted its association with AS. The connection between Lp(a) and AS is primarily due to Lp(a)’s ability to bind to the endothelial surface and infiltrate the inner layers of the aortic valve [26]. Elevated Lp(a) is associated with more rapid progression of aortic valve narrowing [27]. A post-hoc analysis of the FOURIER trial investigating evolocumab highlighted Lp(a) reduction as a potential therapeutic approach for AS [28]. In this analysis, patients receiving evolocumab experienced a lower, though not statistically significant, incidence of new or worsening AS or the need for AVR compared to those on placebo (0.27% vs. 0.41%), with a notable hazard ratio of 0.48 (95% CI, 0.25–0.93). The link between Lp(a) and AVC is particularly promising, raising the potential for new pharmaceutical treatments aimed at lowering Lp(a) to alter the progression of AVC. Emerging treatments, such as antisense oligonucleotides [29, 30] and small interfering RNA [31, 32], have shown significant reductions in Lp(a) levels. Their impact on reducing cardiovascular events is being evaluated in two major clinical trials: the Lp(a)HORIZON Trial [33], expected to conclude in 2025, and the OCEAN(a)-Outcomes Trial [34], expected to finish in 2026. Positive results from these trials could provide the evidence needed to explore whether these new Lp(a)-lowering therapies can non-invasively modify the progression of AVC.

## 8. Other Pathogenetic Mechanisms of AS Progression in DM Patients

As previously discussed, DM triggers and accelerates the onset and progression of AVC through various mechanisms, including hyperinsulinemia, altered lipid metabolism, inflammation and insulin resistance. In the aortic valve, valvular endothelial cells (VECs) provide a protective barrier for underlying tissues, regulating permeability, mediating inflammation, preventing thrombosis, and managing the proliferation of valvular interstitial cells (VICs). Impairment of VEC function due to hyperinsulinemia can promote AVC [4]. Kozakova *et al*. [35] indicates that the combination of hyperinsulinemia and hyperglycemia adversely affects VIC signaling pathways, leading to fibrosis. Furthermore, DM induces glycosylation of lipoproteins, which may result in immune complex formation. These complexes can release growth factors and cytokines, further exacerbating AVC. Valvular cells exposed to high glucose levels increase the expression of inflammatory mediators such as tumour necrosis factor alpha (TNF-α) and interleukin-1 beta (IL-1β), alongside activating several signaling pathways, including protein kinase C (PKC),bone morphogenetic protein (BMP), and transforming growth factor-beta (TGF-β), which contribute to valve remodeling and calcium deposition [36, 37]. Targeting these pathways with specific medications may help slow the development and progression of AVC. DM is also characterized by systemic inflammation, with gut microbiota dysfunction playing a key role. Lipid products from the gut microbiota interact with immune cells, influencing immune responses. In dysbiosis, elevated lipopolysaccharides (LPS) disrupt the intestinal barrier, fostering a pro-inflammatory environment that leads to insulin resistance and hyperglycemia. Conversely, during eubiosis, short-chain fatty acids (SCFA) are produced, which help maintain intestinal barrier integrity, promote immune tolerance, and regulate appetite, providing protective effects. Gut microbiota imbalances can be addressed through dietary modifications and the use of prebiotics, probiotics, and symbiotics. Medications like metformin can modify gut microbiota composition, impacting lipopolysaccharides biosynthesis and short-chain fatty acids metabolism, and alleviate the chronic inflammatory state of DM, potentially delaying the occurrence and progression of AVC [38, 39]. Liraglutide, a glucagon-like peptide 1 (GLP-1) agonist, novel class of medication primarily used to manage type 2 DM, has shown potential as a pharmacological intervention to inhibit AVC progression. In a study [40] involving male *Apoe⁻/⁻* mice, genetically modified to develop atherosclerosis, liraglutide was found to inhibit osteogenic differentiation and inflammation, reduced aortic valve leaflets thickness, and decrease collagen and calcium deposition. work by increasing levels of incretin hormones, which regulate blood sugar levels by promoting insulin secretion and inhibiting glucagon release [41]. Recent research suggests that dipeptidyl peptidase-4 (DPP-4), another class of drugs commonly used to treat DM, may also have cardiovascular benefits, particularly in conditions like AS. Lee *et al*. [42] demonstrated that DPP-4 inhibitors, with favorable pharmacokinetic and pharmacodynamic properties and anti-calcification abilities, were associated with lower degree of increase in maximal transaortic velocity, as well as a reduced frequency of AS progression and AVR.

## 9. Conclusions

The medical management of AS, particularly in patients with type 2 DM, remains a critical and timely topic. While current guidelines set specific goals for LDL-C, there is a gap in understanding how to best control LDL-C in this population. Further studies are needed to clarify the underlying biological mechanism involved in AS progression in DM patients and to improve cardiometabolic management strategies aimed at preventing AS progression in this high-risk group.
